# Suppressor of hepatocellular carcinoma RASSF1A activates autophagy initiation and maturation

**DOI:** 10.1038/s41418-018-0211-7

**Published:** 2018-10-12

**Authors:** Wenjiao Li, Fei Yue, Yuan Dai, Boyun Shi, Guibin Xu, Xianhan Jiang, Xinke Zhou, Gerd P. Pfeifer, Leyuan Liu

**Affiliations:** 1 Center for Translational Cancer Research, Institute of Biosciences and Technology, Texas A&M University, 2121 W. Holcombe Blvd., Houston, TX 77030 USA; 2 The Fifth Affiliated Hospital, Guangzhou Medical University, 510700 Guangzhou, China; 3 Center for Epigenetics, Van Andel Research Institute, Grand Rapids, MI 49503 USA; 4 Department of Molecular and Cellular Medicine, College of Medicine, Texas A&M University, College Station, TX 77843 USA

**Keywords:** Macroautophagy, Tumour-suppressor proteins

## Abstract

RASSF1A (Ras association domain family 1 isoform A) is a tumor suppressor and frequently inactivated by promoter hypermethylation in hepatocellular carcinoma (HCC). Autophagy is to degrade misfolded or aggregated proteins and dysfunctional organelles. Autophagy defects enhance oxidative stress and genome instability to promote tumorigenesis. Activating autophagy flux by increasing levels of the RASSF1A-interacting microtubule-associated protein 1 S (MAP1S) leads to suppression of HCC in addition to extending lifespans. Here we tested whether RASSF1A itself functions as a HCC suppressor and activates autophagy similarly as MAP1S does. We show that RASSF1A deletion leads to an acceleration of diethylnitrosamine-induced HCC and a 31% reduction of median survival times in mice. RASSF1A enhances autophagy initiation by suppressing PI3K-AKT-mTOR through the Hippo pathway-regulatory component MST1 and promotes autophagy maturation by recruiting autophagosomes on RASSF1A-stabilized acetylated microtubules through MAP1S. RASSF1A deletion causes a blockade of autophagy flux. Therefore, RASSF1A may suppress HCC and improve survival by activating autophagy flux.

## Introduction

Hepatocellular carcinoma (HCC) is one of the most common human cancers worldwide and its incidence has tripled in the United States in recent decades [1]. It is the second leading cause of cancer death worldwide [2]. Only 13% of HCC diagnosed in the US are detected early enough to be eligible for curative treatments including surgical resection or liver transplantation, but the prognosis of those patients is poor and the 5-year survival rate is less than 12% [3]. Therefore, there is an urgent need to define the molecular mechanisms underlying HCC development for developing novel therapeutic strategies.

Autophagy, a cellular self-digestion process, functions in the degradation of dysfunctional organelles, misfolded/aggregated proteins and lipid droplets [4]. The anti-apoptotic protein Bcl-2 inhibits autophagy initiation through the PI3K-AKT-mTOR pathway by sequestering Beclin 1 or activates autophagy initiation through the LKB1-AMPK-mTOR pathway by increasing P27 levels [5]. Autophagosomes migrate along tracks made of acetylated microtubules to fuse with lysosomes [6]. Studies of in vivo models highlight the critical role of autophagy in tumor suppression. Mice with deficiency in an autophagy-regulatory protein such as Beclin1, ATG4, ATG5, ATG7 or MAP1S, exhibit a reduction in autophagy activity and an increase in tumorigenesis [7–10]. Studies showed that autophagy defects enhance oxidative stress which trigger DNA double strand breaks (DSB) and genome instability [4, 10, 11], and the deletion of *Beclin 1*, *ATG5*, *ATG7* or *MAP1S* gene in mice was found to promote HCC [4, 8–11].

The Ras association domain family 1 isoform A (RASSF1A) gene locates in the 3p21.3 region of the human genome and was first identified and cloned in 2000 [12]. In HCC, promoter-methylation and *RASSF1A* silencing occurs in over 80% of the patients [13], and deletion of *RASSF1A* in 129S1 mice leads to the formation of liver tumors at late stage of life [14]. RASSF1A was suggested to suppress tumorigenesis through multiple different biological functions including cell cycle arrest, migration inhibition, microtubular stabilization and apoptosis promotion [15–19]. Because of the association of RASSF1A with MST1 and MST2 [20], RASSF1A was suggested to suppress tumorigenesis through the Hippo pathway [21]. However, the exact role and detailed mechanism of RASSF1A in the development of HCC has not been investigated.

Microtubule-associated protein 1S (MAP1S) is a microtubule-associated autophagy activator [22, 23]. MAP1S enhances autophagy initiation through the LKB1-AMPK-mTOR pathway by sustaining the levels of Bcl-2 and P27 [23]. Similar to its homologs, neuronal-specific MAP1A and MAP1B, MAP1S interacts with mammalian autophagy marker LC3 and bridges autophagosomes with microtubules to affect autophagosomal degradation [23]. MAP1S suppresses diethylnitrosamine (DEN)-induced HCC [10, 11]. Natural component spermidine prolongs lifespan and prevents liver fibrosis and HCC by activating MAP1S-mediated autophagy [24]. MAP1S was identified as a major interactive partner of RASSF1A in a yeast two-hybrid analysis of a human liver and brain cDNA library [25, 26]. We reported that MAP1S associates with microtubules stabilized by RASSF1A [19]. The RASSF1A-MAP1S interaction triggered us to hypothesize that RASSF1A may suppress HCC by activating autophagy through MAP1S. Indeed, here we show that RASSF1A stabilizes microtubules by suppressing the activity of RASSF1A-interactive HDAC6, interacts with MAP1S and recruits LC3-II-associated autophagosomes onto acetylated microtubules through MAP1S; and RASSF1A interacts with MST1 and enhances the stability of MST1 to block PI3K-AKT-mTOR pathway, a major pathway suppressing autophagy initiation [27, 28], to promote autophagy initiation. Similar to MAP1S, RASSF1A enhances autophagy initiation and maturation to activate autophagy flux, suppresses oxidative stress, genome instability and DEN-induced HCC, and improves survivals.

## Materials and methods

### Antibodies, plasmids and other reagents

Antibodies against RASSF1A for immunoblots of HeLa cells (ab23950) and hepatocytes (ab97749) were from Abcam. Antibody against RASSF1A (14-6888-82) for immunoprecipitation was from eBioscience. The siRNAs specific to human RASSF1A (SC-44070), normal mouse control IgG (SC-2025), normal rabbit control IgG (SC-2027), and antibodies against GAPDH (SC-25778), acetylated-α-tubulin (SC-23950), HDAC6 (SC-11420), GFP (SC-8334), Bcl-2 (SC-7382), P27 (SC-528), AKT (SC-5298) were from Santa Cruz Biotechnology. Negative control siRNA (AM4635) were from Invitrogen. Antibodies against Myc-Tag (2276), HA-Tag (3724), p-AKT (2965 S), p-MST1 (3681 S), p-S6K (9205 S) were from Cell Signaling Technology. Antibody against MST1 (22245-1-AP) was from Proteintech. Chloroquine (CQ, C6628), Hematoxylin and Eosin (H&E, HT110116), and antibody against Flag (F3165) were from Sigma. Trypsin (CA014) was from GenDEPOT. Other reagents not mentioned here were described by Li et al. and Yue et al. [24, 29]. Plasmids encoding Myc-LC3 (#24919), HDAC6 (#30482) and P27 (#14049) were purchased from Addgene. The construction of GFP-RASSF1A, HA-RASSF1A, RFP-LC3, GFP-LC3, HA-MAP1S isoforms (HA-FL, HA-HC, HA-SC and HA-LC), GFP-MAP1S full length, HA-HBD (R653-Q855 fragment of MAP1S in HA-PCMV plasmid), HA-FLΔ (HA-MAP1S with R653-Q855 fragment deleted) were described previously [19, 25, 30].

### Animal experiments

Animal protocols were approved by the Institutional Animal Care and Use Committee, Institute of Biosciences and Technology, Texas A&M Health Science Center. All animals received humane care according to the criteria outlined in the “Guide for the Care and Use of Laboratory Animals” prepared by the National Academy of Sciences and published by the National Institutes of Health (NIH publication 86–23 revised 1985). Wild-type (RASSF1A^+/+^) and RASSF1A knockout mice (RASSF1A^−/−^) in C57BL/6 J background were bred and genotyped as described [31]. Male littermates of wild-type and RASSF1A^−/−^ mice at 15-day-old were intraperitoneally injected with a single dose of diethylnitrosamine (DEN) to induce HCC. Liver tissues were harvested immediately after the animals were euthanized and then frozen or fixed for immunoblotting, immunostaining, H&E staining and oxidative stress analysis similarly as we previously described [10, 29]. The RASSF1A mRNA levels in liver tissues were quantified by real-time PCR using primers RASSF1A Forward (5′-GTACAACACGCAATCCGTC-3′), RASSF1A Reverse (5′-GCAGACGAGC GCGCGAC-3′), β-actin Forward (5′-GCACCAGGGTGTGATGGTG-3′), and β-actin Reverse (5′-TGGATGGCTACGTACATGGC-3′). In addition, mice for survival analysis were injected with DEN at 15 days after birth and observed to record their survival times when they were found dead or when they were found to be moribund. The Kaplan-Meier method was used to analyze the overall survival and median survival times.

### Plasmid construction

Four fragments of RASSF1A (F1, F2, F3 and F4) were amplified by PCR using HA-RASSF1A as a template. Amplified fragments were digested with *XhoI and BamHI* and ligated with pEGFP-C3 vector digested with *XhoI and BamHI* similarly as we previously reported [25]. The pair of primers for amplifying F1 (fragment from amino acid 1–151) is F1 Forward (5′-CCGCTCGAGATGTCGGGGGAGCCTGAGCTCATT-3′) and F1 Reverse (5′-CGCGGATCCTCAGAAGAGGTTGCTTTGATCTGGGC-3′); F2 (fragment from amino acid152–186) F2 Forward (5′-CCGCTCGAGATGAGCTTGAACAAGGACGGTTC-3′) and F2 Reverse (5′-CGCGGA TCCTCACTGCAAGGAGGGTGGCTTCTT-3′); F3 (fragment from amino acid 187–287) F3 Forward (5′-CCGCTCGAGGATGCCCGGCGGGGCCCA GGA-3′) and F3 Reverse (5′-CGCGGATCCTCAGTCATTTTCCTTCAGGACAAA GCTC-3′); and F4 (fragment from amino acid 288–340) F4 Forward (5′-CCGCTCGAGTCTGGGGAGGTGAACTGGGA-3′) and F4 Reverse (5′-CGCGGAT CCTCACCCAAGGGGGCAGGCGT-3′). Primers 5′-CCGCTCGAGTCTGGGGAGGTGAACTGGGA-3′ and 5′-CGCGGAT CCTCACCCAAGGGGGCAGGCGT-3′ and template HA-RASSF1A were used to delete RA domain (from amino acid 187–287) from RASSF1A to generate RAΔ construct as we previously described [30].

To establishment of RASSF1A knockout HeLa cell line by CRISPR/Cas9, guide RNAs targeting human RASSF1A gene were designed using Optimized Crispr Design (http://crispr.mit.edu/). Synthesized DNA oligos were inserted into crispr/cas9 vector pSpCas9(BB)−2APuro (PX459) (Addgene, #48139). HeLa cells were transiently transfected with a pool of three plasmids encoding Cas9 nuclease and guide RNAs targeting for RASSF1A or the vector for wild-type control. The sequences of three pairs of DNA oligos for gRNAs are 1) RASSF1A g1-F: 5′-CACCGAACGCGCTGCGCATCGCGCG-3′, RASSF1A g1-R: 5′-AAACCGCGCGATGCGCAGCGCGTTC-3′; 2) RASSF1A g2-F: 5′-CACCGCAACGCGCTGCGCATCGCGC-3′, RASSF1A g2-R: 5′-AAACGCGCGATGCGCAGCGCGTTGC-3′; and 3) RASSF1A g3-F: 5′-CACCGTCGCACCACGTGTGCGTGGC-3′, RASSF1A g3-R: 5′-AAACGCCACGCACACGTGGTGCGAC-3′.

### Cell culture and isolation of primary mouse hepatocytes and mouse embryonic fibroblasts (MEFs)

Cell lines including HeLa, human embryonic kidney (HEK)−293T, HeLa cells stably expressing ERFP-LC3 (HeLa-RFP-LC3), and MEFs were established as described [23]. Cell lines obtained from the American Type Culture Collection (Manassas, VA, USA) were cultured in DMEM containing 10% FBS and antibiotics. Cell lines used in this study were authenticated by ATCC but not further confirmed because they were solely used for biochemical and cell biological assays and not for study of cancer biological functions. Mouse primary hepatocytes were isolated from 12-week-old male mice by the two-steps liver perfusion method as previously described [24]. Cell transfection, immunoblotting, immunoprecipitation, in vitro microtubular assembling, H&E staining, and confocal fluorescent microscopy were performed as previously described [23, 29, 30].

## Results

### RASSF1A suppresses DEN-induced HCC and maintains mouse survivals

**Fig. 1:**
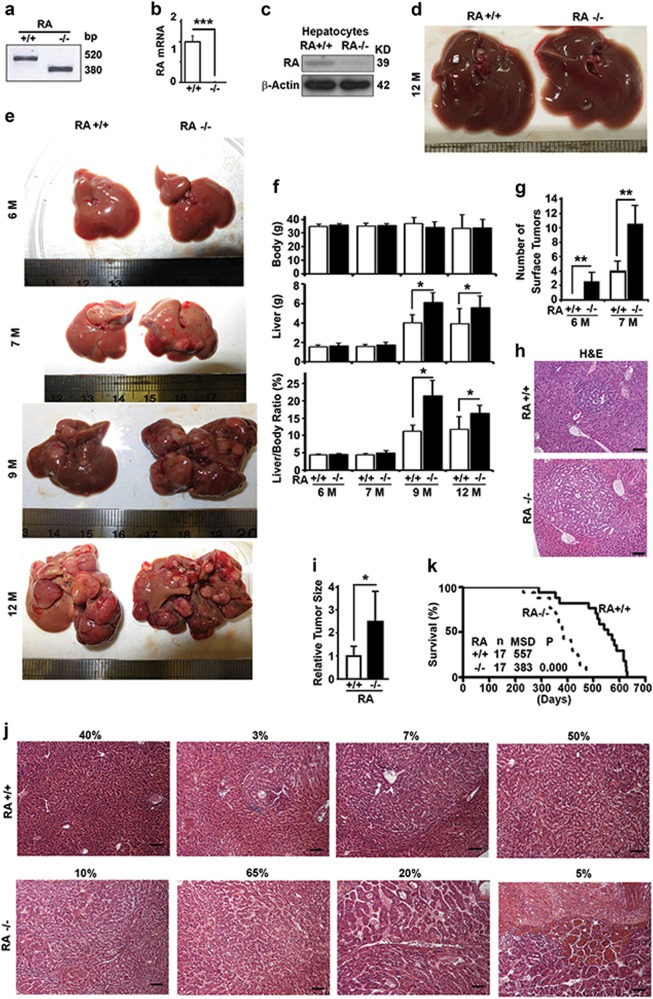
RASSF1A suppresses hepatocarcinogenesis and promotes survival in DEN-treated mice. **a** PCR analysis of DNA samples from mouse tails to genotype wild-type (RA+/+) and RASSF1A^−/−^ mice (RA−/−). **b** Quantitative real-time PCR analysis of the levels of RASSF1A mRNA in liver tissues from wild-type and RASSF1A^−/−^ mice. **c** Representative immunoblot showing levels of RASSF1A protein in hepatocytes isolated from wild-type and RASSF1A^−/−^ mice. **d** Representative images of liver tissues from 12-month-old untreated wild-type and RASSF1A^−/−^ mice in normal conditions. **e** Representative images of liver tissues from DEN-treated wild-type and RASSF1A^−/−^ mice at different ages. **f** Plots of body weights, liver weights, ratios of body weight to liver weight of mice as shown in **e**. **g** Plots of number of surface tumors of mice as shown in **e**. **h** Comparative H&E staining among the liver tissues from DEN-treated 6-month-old mice described in **e**. Bar = 20 µm. **i** Plots of tumor size as shown in **h**. **j** Representative images showing different types of H&E staining of liver tissues from DEN-treated 12-month-old mice as shown in **e**. Bar = 20 µm. The percentage shown on top of each panel is the relative frequency of the type. **k** The Kaplan–Meier survival curves showing the survival times of male littermates of wild-type and RASSF1A^−/−^ mice treated with DEN. n number of mice, MSD median survival days. The significance of difference between two groups was estimated by log-rank test and p value was the probability larger than the *χ*^2^ value. Here and later, all experiments were repeated at least three times. ns, not significant or *p* > 0.05; *p ≤ 0.05; **p ≤ 0.01; and ***p ≤ 0.001

To investigate the function of RASSF1A in HCC, we imported RASSF1A^−/−^ mice as a gift and identified them by genotyping with DNA samples from tails (Fig. 1a) [31]. The *RASSF1A* gene was confirmed to be completely deleted in liver tissues by RT-PCR analysis of mRNA (Fig. 1b). Although not successful with samples from mouse liver tissues and other tissues, we identified a RASSF1A antibody that was able to detect a weak band of RASSF1A in immunoblots with samples made from primary cultured mouse hepatocytes (Fig. 1c). We maintained the mice under identical conditions for 12 months and were unable to detect any tumor on the liver surface (Fig. 1d). We started to inject 15 days-old male mice with a single dose of DEN, a widely-used chemical carcinogen to induce HCC in mice [32]. We started to observe small tumor foci on the surfaces of livers of RASSF1A^−/−^ mice at 6 months and wild-type mice at 7 months after DEN treatment (Fig. 1e). At this stage, although there was no difference in body and liver weights (Fig. 1e, f), RASSF1A^−/−^ mice developed more tumor foci as observed on their liver surface (Fig. 1e, g) and larger tumor foci as shown in liver tissue sections (Fig. 1h, i). When mice became older, individual tumors were amplified so that large tumor foci occupied the entire liver surfaces of RASSF1A^−/−^ mice at 12 months (Fig. 1e), resulting in a significant higher liver weights and liver/body weight ratios than the wild-type (Fig. 1e, f). Examining the liver tissue sections from 12 months-old mice in detail revealed that wild-type mice contained about 40% area with normal liver structures, 10% with encircled tumor foci and 50% area with typical HCC trabecular structure, while RASSF1A^−/−^ mice contained only 10% area with encircled tumor foci and small portion of normal liver structure, 65% typical HCC trabecular structure and 25% highly distorted liver structures (Fig. 1j). Due to the accelerated hepatocarcinogenesis, RASSF1A deletion resulted in a 31% reduction in mouse median survival times (Fig. 1k). Therefore, RASSF1A suppresses DEN-induced hepatocarcinogenesis and sustains mouse survivals.

### RASSF1A suppresses oxidative stress and DNA double strand breakage

**Fig. 2:**
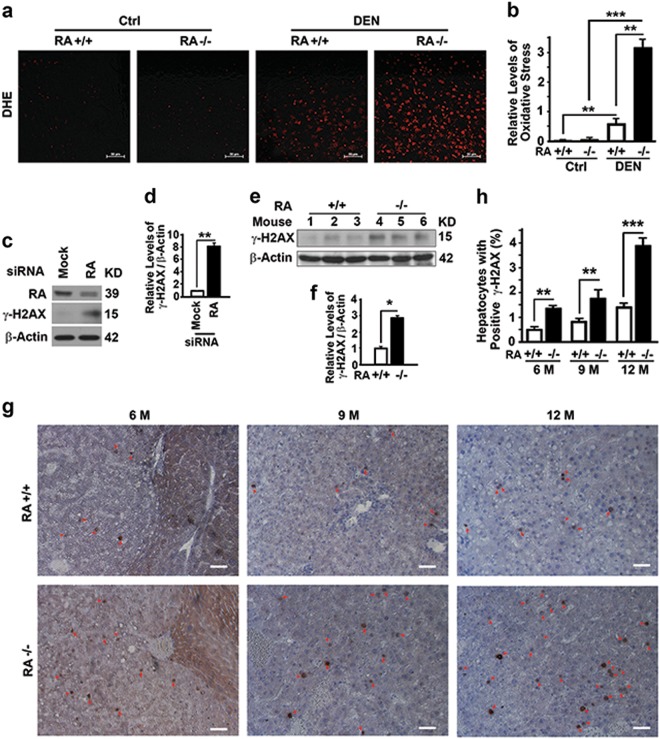
RASSF1A suppresses oxidative stress and DNA damage in mouse liver tissues. **a**, **b** Representative images (**a**) and quantification (**b**) showing levels of oxidative stress revealed by dihydroethidine hydrochloride (DHE) staining in liver tissues from wild-type and RASSF1A^−/−^ mice treated with vehicle (Ctrl) or DEN for two days. Bar = 50 µm. **c**, **d** Representative immunoblot (**c**) and quantification (**d**) showing γ-H2AX levels in HeLa cells treated with random (Mock) or RASSF1A-specific siRNA (RA). **e**, **f** Representative immunoblot (**e**) and quantification (**f**) showing γ-H2AX levels in liver tissues from DEN-treated 6-month-old wild-type and RASSF1A^−/−^ mice. **g** Representative immunostaining of γ-H2AX in liver tissue sections from DEN-treated wild-type and RASSF1A^−/−^ mice as described in (Fig. 1e). Red arrows indicate γ-H2AX positive cells. Bar = 10 µm. **h** Plots of percentage of γ-H2AX positive cells to total cells in liver sections as shown in **g**

Oxidative stress induces DNA double-strand breakage (DSB) and genome instability through cycles of cell division to promote tumorigenesis [4, 10, 33]. There was not much difference in levels of oxidative stress in liver tissues between wild-type and RASSF1A^−/−^ mice when untreated, as measured by dihydroethidine hydrochloride (DHE) staining. However, RASSF1A deletion induced high levels of oxidative stress at two days after DEN injection (Fig. 2a,b). RASSF1A suppression caused increases of γ-H2AX levels representing DNA DSB in HeLa cells (Fig. 2c, d) and mouse liver tissues collected at 6 months after DEN injection (Fig. 2e, f). Immunostaining also confirmed that RASSF1A deletion promoted DNA DSB (Fig. 2g, h). Therefore, RASSF1A suppresses oxidative stress and genome instability.

### RASSF1A enhances autophagy flux

**Fig. 3:**
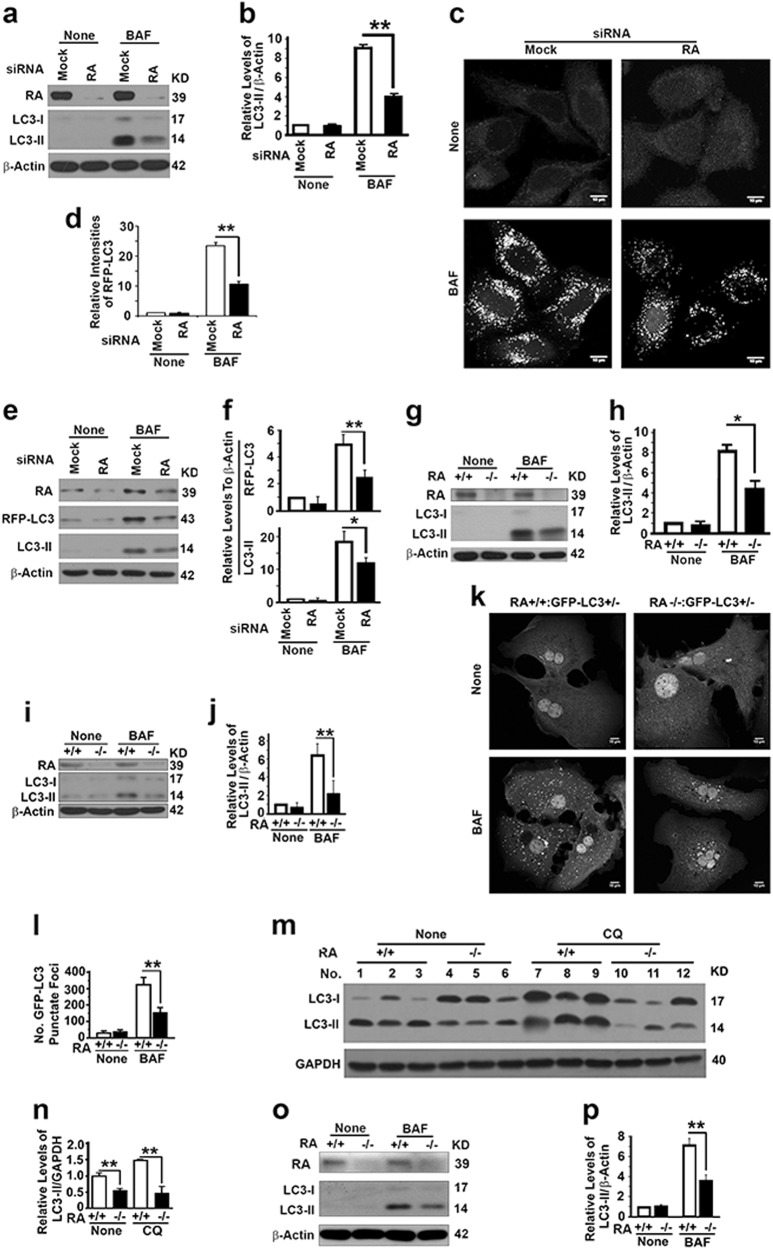
RASSF1A activates autophagy flux. **a**, **b** Representative immunoblot (**a**) and quantification (**b**) showing LC3-II levels in HeLa cells treated with random (Mock) or RASSF1A-specific siRNAs (RA) in the absence (None) or presence of lysosomal inhibitor BAF (10 µM overnight before harvest). **c**, **d** Representative images (**c**) and quantification (**d**) showing the number of RFP-LC3 punctate foci in HeLa cells stably expressing RFP-LC3 treated with random (Mock) or RASSF1A-specific siRNAs (RA) in the absence (None) or presence of BAF. Bar = 10 µM. **e**, **f** Representative immunoblots (**e**) and quantification showing the levels of exogenous RFP-LC3 and endogenous LC3-II (**f**) in similar cells as shown in **c**. **g**, **h** Representative immunoblot (**g**) and quantification (**h**) showing LC3-II levels in wild-type and RASSF1A^−/−^ MEFs in the absence (None) or presence of BAF. **I**, **j** Representative immunoblot (**i**) and quantification (**j**) showing LC3-II levels in hepatocytes similarly isolated from mice shown in **g**. **k**, **l** Representative images (**k**) and quantification (**l**) showing the number of GFP-LC3 punctate foci in hepatocytes isolated from GFP-LC3 transgenic wild-type and RASSF1A^−/−^ mice in the absence (None) or presence of BAF. Scale bar, 10 µM. **m**, **n** Representative immunoblot (**m**) and quantification (**n**) showing LC3-II levels in liver tissues from wild-type and RASSF1A^−/−^ mice injected with saline (None) or lysosomal inhibitor CQ. **o**, **p** Representative immunoblot (**o**) and quantification (**p**) showing LC3-II in hepatocytes isolated from 4-month old DEN-treated wild-type and RASSF1A^−/−^ mice in the absence or presence of BAF

Because of close relation between autophagy and tumorigenesis and because the RASSF1A-interactive protein MAP1S is involved in autophagy regulation and tumor suppression [10, 19, 22–24, 34], we reasoned that RASSF1A regulates autophagy and suppresses tumorigenesis. We first suppressed the expression of RASSF1A with specific siRNAs in HeLa cells and found that the autophagy flux reflected by the LC3-II levels in the presence of lysosomal inhibitor bafilomycin A1 (BAF) was significantly reduced (Fig. 3a, b). Such reduction in autophagy flux was confirmed by the reduction in the number of RFP-LC3 punctate foci representing autophagosomes (Fig. 3c–f). We further investigated the impact of RASSF1A on autophagy flux and confirmed that RASSF1A deletion resulted in reductions of LC3-II levels in BAF-treated MEFs (Fig. 3g, h) and hepatocytes (Fig. 3i, j) isolated from mice, number of GFP-LC3 punctate foci in BAF-treated hepatocytes isolated from GFP-LC3 transgenic mice (Fig. 3k, l), and LC3-II levels in liver tissues treated with another lysosomal inhibitor CQ (Fig. 3m, n). Similarly to the case in healthy livers, RASSF1A deletion resulted in a reduction in LC3-II levels in hepatocytes isolated from 4-month old DEN-treated livers carrying premalignant lesions (Fig. 3o, p). All results show that RASSF1A activates autophagy.

### RASSF1A does not regulate autophagy initiation through MAP1S-Bcl-2-P27 pathway

**Fig. 4:**
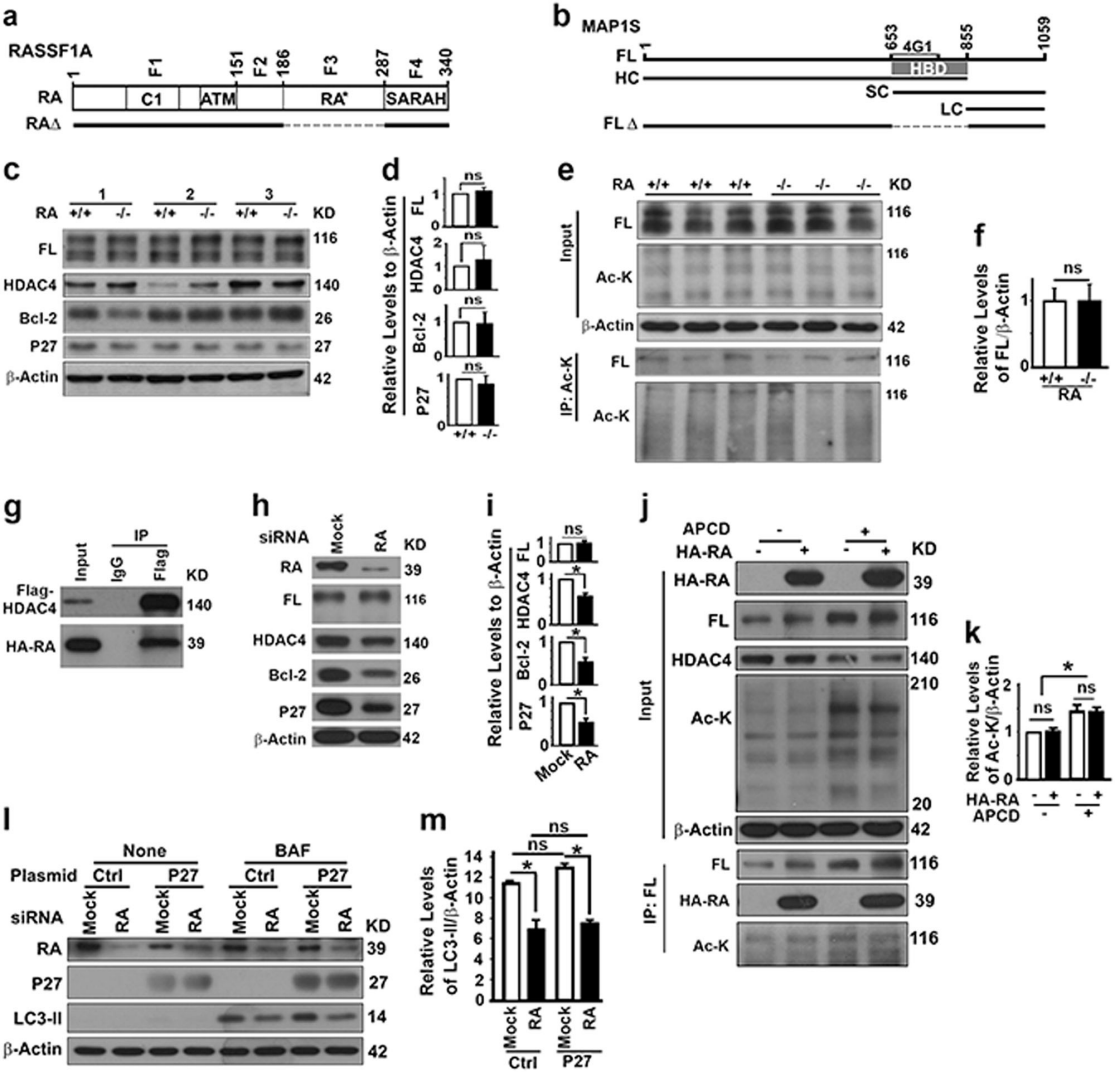
RASSF1A regulates autophagy initiation not through MAP1S. **a** A diagram showing the domain structures of RASSF1A protein and its mutant constructs. RASSF1A has four characterized domains: C1, phorbol ester/diacylglycerol binding domain; ATM, ataxia-telangiectasia mutated domain; RA*, Ras-association (RalGDS/AF-6) domain (F3); SARAH, MST and SAV1 binding domain (F4). Four fragments of RASSF1A (F1–4) were fused with GFP, respectively. RASSF1A with RA domain deleted (RAΔ) was fused with HA. **b** A diagram showing the domain structures of MAP1S protein and its mutant constructs. FL full length, HC heavy chain, SC short chain, LC light chain, 4G1 region recognized by MAP1S monoclonal antibody 4G1, HBD HDAC4‐binding domain (R653‐Q855), FLΔ full length MAP1S with HBD domain deleted. **c**, **d** Representative immunoblots (**c**) and quantification (**d**) showing the impact of RASSF1A on levels of MAP1S, HDAC4, Bcl-2 and P27 in liver tissues from 3 pairs of wild-type and RASSF1A^−/−^ littermates. **e**, **f** Representative immunoblots (**e**) and quantification (**f**) showing the impact of RASSF1A on levels of acetylated MAP1S in liver tissues from wild-type and RASSF1A^−/−^ littermates. Lysates were precipitated with Ac-K antibody and blotted with MAP1S-specific 4G1 antibody and Ac-K antibody. **g** Representative immunoblots showing the interaction of HDAC4 with RASSF1A in 293T cells transiently expressing Flag-HDAC4 and HA-RASSF1A. **h**, **i** Representative immunoblots (**h**) and quantification (**i**) showing the impact of RASSF1A depletion on levels of MAP1S, HDAC4, Bcl-2 and P27 in HeLa cells treated with random (Mock) or RASSF1A-specific siRNAs (RA). **j**, **k** Representative immunoblots (**j**) and quantification (**k**) showing the impact of overexpressed RASSF1A on levels of acetylated MAP1S in HeLa cells overexpressing HA-RASSF1A in the absence or presence of HDAC4 inhibitor apicidin (APCD). Lysates were precipitated with MAP1S-specific 4G1 antibody and blotted with Ac-K antibody and 4G1 antibody. **l**, **m** Representative immunoblots (**l**) and quantification (**m**) showing whether elevated levels of P27 affect the impact of RASSF1A on LC3-II levels in HeLa cells treated with random (Mock) or RASSF1A-specific siRNAs (RA) and transiently transfected with empty control vector or vector encoding P27 in the absence or presence of BAF

Deleting either MAP1S [23, 29] or RASSF1A (Fig. 3) leads the same reduction in autophagy flux. We reasoned that RASSF1A may regulate autophagy initiation through its interactive protein MAP1S (Fig. 4a, b). MAP1S regulates Bcl-2 and P27 to control autophagy flux through the LKB1-AMPK-mTOR pathway [23] and itself is regulated by HDAC4 [30]. RASSF1A deletion did not alter the levels of MAP1S, HDAC4, Bcl-2 and P27 (Fig. 4c, d), and the levels of acetylated MAP1S (Fig. 4e, f). RASSF1A interacted with HDAC4 in HeLa cells (Fig. 4g). The levels of MAP1S remained constant while levels of HDAC4, Bcl-2 and P27 did reduce when RASSF1A was silenced (Fig. 4h, i). However, levels of acetylated MAP1S were not changed by RASSF1A overexpression either in the absence or presence of HDAC4 inhibitor apicidin (Fig. 4j, k). Forced expression of P27 did not restore the reduced autophagy flux in HeLa cells carrying reduced levels of P27 (Fig. 4l, m). Therefore, RASSF1A regulates autophagy initiation not through the MAP1S-Bcl-2-P27 non-canonical pathway [23].

### RASSF1A suppresses PI3K-AKT-mTOR pathway to promote autophagy initiation through Hippo pathway regulatory protein MST1

**Fig. 5:**
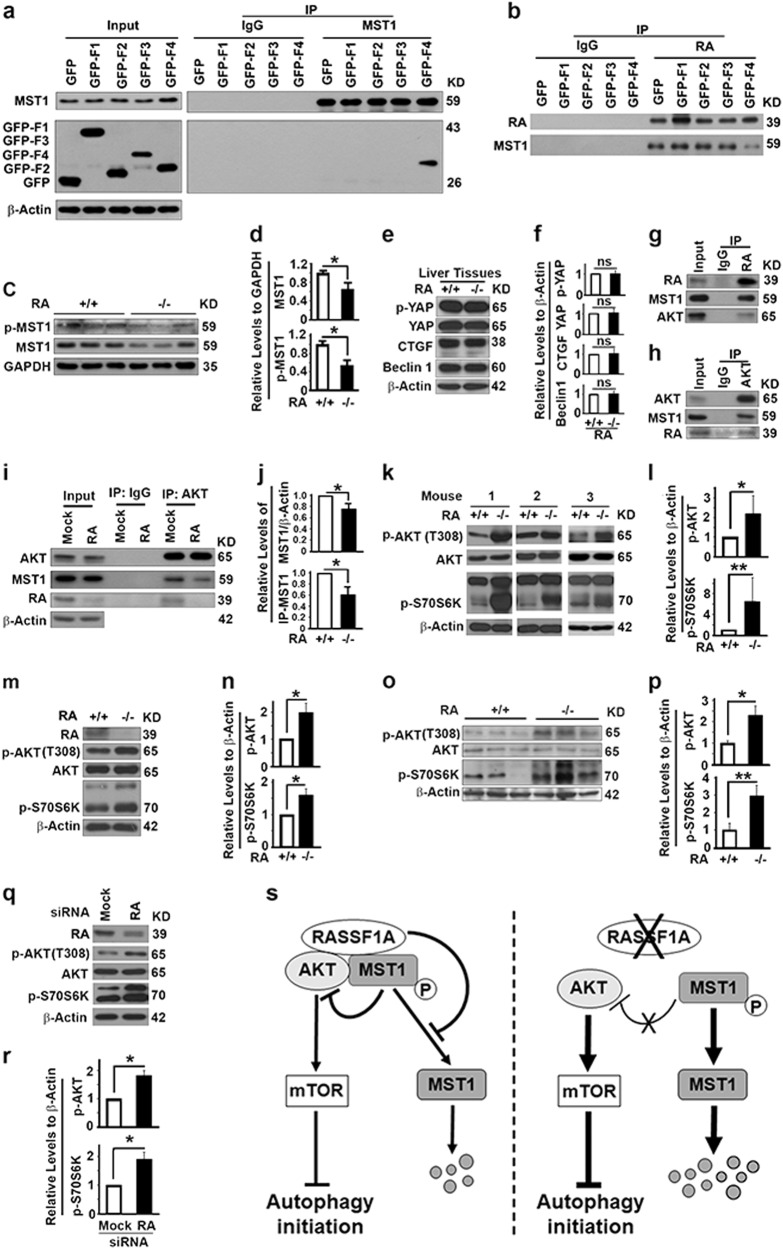
RASSF1A suppresses PI3K-Akt-mTOR pathway to promote autophagy initiation through Hippo pathway regulatory protein MST1. **a** Representative immunoblots showing the fragment of RASSF1A coimmunoprecipitated with MST1 in 293T cells. **b** Representative immunoblots showing the impact of fragments of RASSF1A on the amount of MST1 coimmunoprecipitated with RASSF1A in 293T cells. Inputs are the same as in panel **a**. **c**, **d** Representative immunoblots (**c**) and quantification showing the impact of RASSF1A on levels of total and p-MST1 (**d**) in mouse liver tissues. **e**, **f** Representative immunoblots (**e**) and quantification (**f**) showing the impact of RASSF1A on levels of p-YAP, YAP, CTGF and Beclin 1 in mouse liver tissues. **g**, **h** Representative immunoblots showing levels of MST1 and Akt coimmunoprecipitated with RASSF1A (**g**) or levels of MST1 and RASSF1A coimmunoprecipitated with Akt (**h**) in HeLa cells. **I**, **j** Representative immunoblots (**i**) and quantification showing the impact of RASSF1A suppression on the levels of total MST1 or MST1 coimmunoprecipitated (IP-MST1) with Akt (**j**) in HeLa cells treated with random (Mock) or RASSF1A-specific siRNAs. **k**–**r** Representative immunoblots (**k**, **m**, **o**, **q**) and quantification showing the levels of p-AKT and p-S70S6K (**l**, **n**, **p**, **r**) in mouse liver tissues (**k**, **l**), MEFs (**m**, **n**), liver tissues from DEN-treated 6-month-old mice (**o**, **p**), or HeLa cells treated with random (Mock) or RASSF1A-specific siRNAs (**q**, **r**). **s** A diagram showing the mechanism by which RASSF1A promotes autophagy initiation through MST1-AKT-mTOR pathway

Since RASSF1A maintains the stability and activity of MST1 (mammalian STE20-like kinase 1) by directly interacting with MST1 and preventing it from dephosphorylation by PP2A, RASSF1A was suggested to be an important regulator of the Hippo pathway [20]. We confirmed the interaction of RASSF1A with MST1 specifically through its SARAH domain (F4) (Fig. 5a). The overexpressed SARAH domain competed with endogenous RASSF1A to bind with MST1, leading to a reduction in amounts of RASSF1A-associated endogenous MST1 (Fig. 5b). Then, we examined the impact of RASSF1A deletion on the components of the Hippo pathway. RASSF1A depletion did cause reductions in levels of total and phosphorylated MST1 (Fig. 5c, d) but had no impact on levels of p-YAP, YAP and CTGF in mouse liver tissues (Fig. 5e, f), suggesting RASSF1A does not impact much the downstream effectors of the Hippo pathway at least in liver tissues. Since MST1 was reported to interact with Beclin 1 and Bcl-2 to prevent autophagy initiation in cardiomyocytes [35], the reduction in levels of MST1 due to RASSF1A deletion was predicted to cause an activation of autophagy initiation, which is contradicted with the observed autophagy inhibition in liver tissues (Fig. 3). We found that RASSF1A deletion did not alter the levels of Bcl-2 (Fig. 4c, d) and Beclin 1 (Fig. 5e, f) in liver tissues. Therefore, the impact of RASSF1A on autophagy initiation does not work through Beclin 1 and Bcl-2.

It was also reported that MST1 interacts with AKT and acts as a direct inhibitor of AKT [36]. We reasoned that RASSF1A may act on the PI3K-AKT-mTOR pathway through MST1 to regulate autophagy initiation. We found that MST1 and AKT were co-immunoprecipitated with RASSF1A (Fig. 5g) and MST1 and RASSF1A were co-immunoprecipitated with AKT (Fig. 5h), suggesting RASSF1A, AKT and MST1 form a protein complex. RASSF1A depletion led to a reduction in levels of total MST1 so that the levels of MST1 co-immunoprecipitated with AKT were significantly reduced in RASSF1A-suppressed HeLa cells (Fig. 5i, j). RASSF1A depletion caused increases in levels of phosphorylated AKT and phosphorylated S70S6K, a downstream effector of mTOR, in mouse liver tissues (Fig. 5k, l), MEFs (Fig. 5m, n) and DEN-treated 6-month-old mouse liver tissues (Fig. 5o, p), as well as HeLa cells (Fig. 5q, r). Therefore, RASSF1A depletion causes the dephosphorylation and instability of MST1, leading to a reduction in MST1 and AKT interaction and thereby activation of the PI3K-AKT-mTOR pathway to inhibit autophagy initiation (Fig. 5s).

### RASSF1A interacts with HDAC6 to enhance acetylation of α-tubulin and co-localizes with acetylated microtubules

**Fig. 6:**
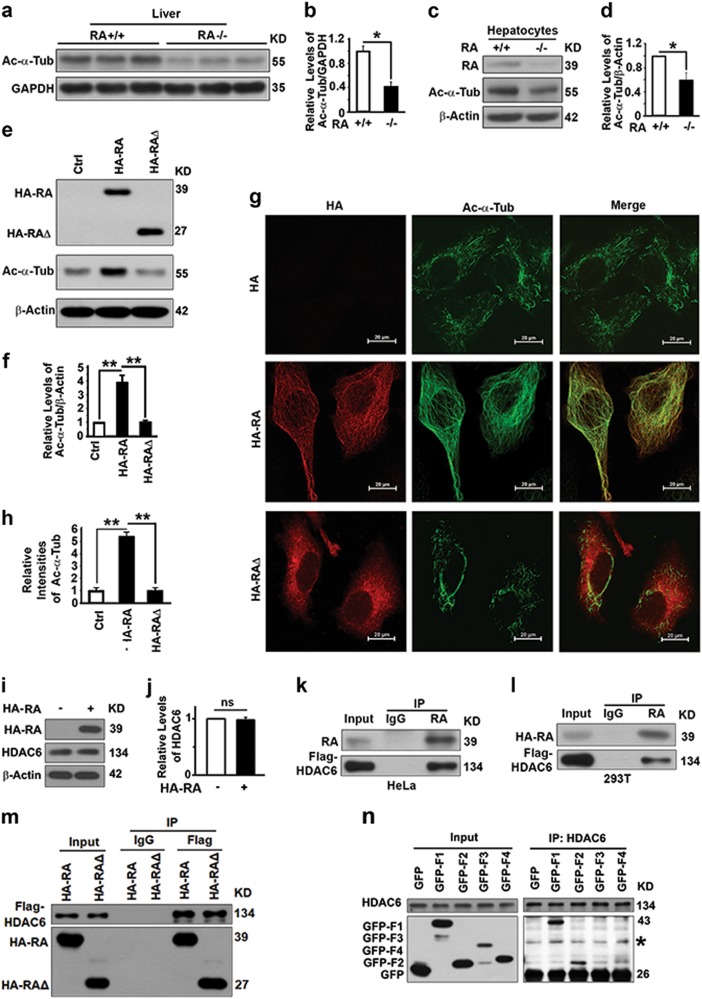
RASSF1A interacts with HDAC6 to enhance acetylation of α-Tubulin and co-localizes with acetylated microtubules. **a**, **b** Representative immunoblot (**a**) and quantification (**b**) showing levels of acetylated α-tubulin (Ac-α-Tub) in liver tissues from 4-month-old wild-type and RASSF1A^−/−^ mice. **c**, **d** Representative immunoblot (**c**) and quantification (**d**) showing levels of acetylated α-tubulin in hepatocytes isolated from wild-type and RASSF1A^−/−^ mice. **e**, **f** Representative immunoblot (**e**) and quantification (**f**) showing levels of acetylated α-tubulin in HeLa cells transiently transfected with control plasmid, plasmid expressing HA-RASSF1A (HA-RA), and plasmid expressing HA-RASSF1A∆ (HA-RA∆). **g**, **h** Representative images (**g**) and quantification (**h**) showing the immunostaining intensities of acetylated α-tubulin in cells similar to those in **e**. Bar = 20 µm. Red, HA-RA or HA-RA∆; green, acetylated a-tubulin; and yellow, colocalization. **i**, **j** Representative immunoblot (**i**) and quantification (**j**) showing the impact of RASSF1A on levels of HDAC6 in cells similar to those in **e**. **k**, **l** Representative immunoblots showing levels of Flag-HDAC6 coimmunoprecipitated with endogenous RASSF1A from HeLa cells (**k**) or exogenous RASSF1A in 293T cells (**l**) transiently overexpressing Flag-HDAC6. **m** Representative immunoblots showing levels of RA-HA or RA-RA∆ coimmunoprecipitated with Flag-HDAC6 in 293T cells. **n** Representative immunoblot showing levels of GFP fused fragments of RASSF1A coimmunoprecipitated with HDAC6 from lysates of HeLa cells transiently expressing GFP fused RASSF1A constructs

To further understand the molecular mechanism by which RASSF1A activates autophagy flux, we tested the impact of RASSF1A on the levels of acetylated α-tubulin from which stable acetylated microtubules are assembled to support trafficking of autophagosomes to fuse with lysosomes [6]. RASSSF1A deletion led to a reduction in levels of acetylated α-tubulin in mouse liver tissues (Fig. 6a, b) and their derived hepatocytes (Fig. 6c, d), while overexpressed RASSF1A caused increases in levels of acetylated α-tubulin (Fig. 6e, f) and an enhancement of stable acetylated microtubules in HeLa cells (Fig. 6g, h). Although overexpressed RASSF1A did not decrease levels of HDAC6 that regulates acetylation of α-tubulin (Fig. 6i, j) [37], it interacted with HDAC6 (Fig. 6k, l). Such interaction may lead to an impairment of HDAC6 deacetylase activity and promotion of α-tubulin acetylation as reported [15]. RA domain is required for the association of RASSF1A with hyperstabilized microtubules [19]. RA∆, a RASSF1A mutant with the RA domain deleted (Fig.4a), also interacted with HDAC6 (Fig. 6m), but had no impact on the acetylation of microtubules (Fig. 6e–h). RASSF1A interacted with HDAC6 mainly through F1 and secondly through F2 (Fig. 6n). Therefore, RASSF1A binds with HDAC6 through F1 and F2 to suppress its activity to promote microtubular acetylation through its RA domain.

### RASSF1A helps recruit autophagosomes onto acetylated microtubules through LC3-interactive MAP1S and regulates autophagy initiation and autophagosomal degradation

**Fig. 7:**
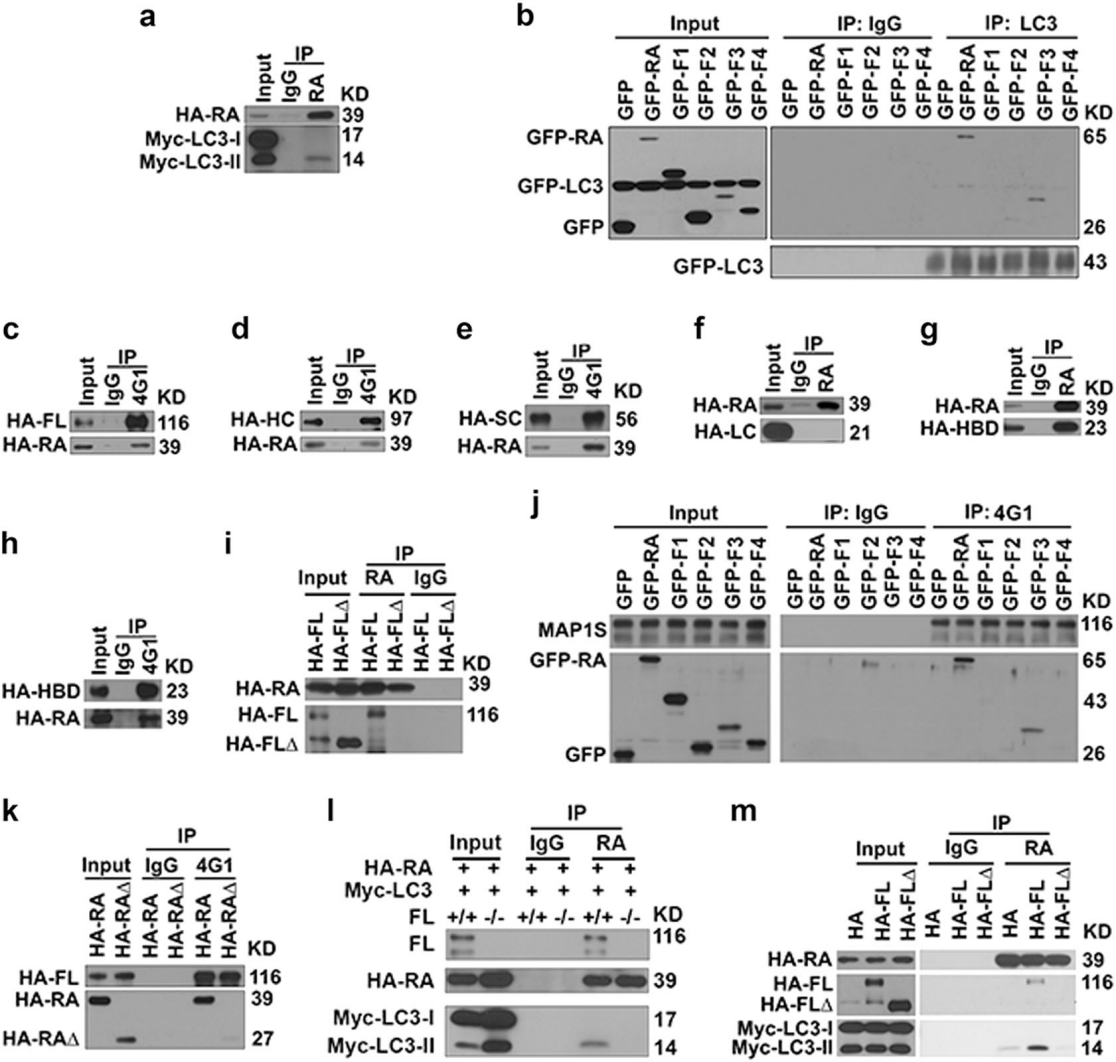
There are specific interactions among RASSF1A, MAP1S and LC3. **a** Representative immunoblot showing levels of Myc-LC3-II coimmunoprecipitated with HA-RASSF1A from lysates of MEFs transiently expressing HA-RASSF1A and Myc-LC3. **b** Representative immunoblot showing levels of GFP fused fragments of RASSF1A coimmunoprecipitated with LC3 from lysates of 293T cells transiently expressing GFP-LC3 and GFP fused RASSF1A constructs. **c**–**e** Representative immunoblots showing levels of RASSF1A coimmunoprecipitated with FL (**c**), HC (**d**), SC (**e**). **f**, **g** Representative immunoblots showing levels of LC (**f**) and HBD (**g**) coimmunoprecipitated with RASSF1A. **h** Representative immunoblots showing levels of RASSF1A coimmunoprecipitated with HBD in 293T cells transiently expressing HA-RASSF1A and HA-HBD. MAP1S-specific antibody 4G1 recognizes HBD. **i** Representative immunoblots showing levels of FL or FL∆ of MAP1S coimmunoprecipitated with RASSF1A in 293T cells transiently expressing HA-RASSF1A and HA-FL or HA-FL∆. **j** Representative immunoblots showing levels of RASSF1A constructs coimmunoprecipitated with endogenous MAP1S in 293T cells transiently expressing GFP-tagged RASSF1A constructs. **k** Representative immunoblots showing levels of RASSF1A or RA∆ coimmunoprecipitated with FL of MAP1S in 293T cells transiently expressing HA-FL and HA-RA or HA-RA∆. **l** Representative immunoblots showing the impact of MAP1S on levels of Myc-LC3-II coimmunoprecipitated with RASSF1A in wild-type and MAP1S^−/−^ MEFs transiently expressing HA-RASSF1A and Myc-LC3-II. **m** Representative immunoblots showing levels of FL or FL∆ of MAP1S and Myc-LC3-II coimmunoprecipitated with RASSF1A in 293T cells transiently expressing HA-RASSF1A, Myc-LC3 and HA-fused FL or FLΔ

We reported that RASSF1A stabilizes microtubules and helps recruit MAP1S on the stabilized microtubules and MAP1S is required for LC3 to associate with RASSF1A-stabilized microtubules [23, 38]. RASSF1A specifically interacted with LC3-II through its RA domain (F3) (Figs. 4a, 7a, b). RASSF1A interacted with full-length (FL), heavy chain (HC) and short chain (SC) but not light chain (LC) of MAP1S (Figs. 4b, 7c–f), predicting that the HDAC4-binding domain (HBD) of MAP1S mediates the interaction with RASSF1A. Such prediction was confirmed by the facts that HBD interacted (Fig. 7g, h) while full length MAP1S with HBD deleted (FL∆) did not interact with RASSF1A (Fig. 7i). The RA domain of RASSF1A (Fragment F3) interacted with MAP1S (Fig. 7j) and deleting the RA domain in RASSF1A (RA∆) abolished its interaction with MAP1S (Fig. 7k). The interaction of RASSF1A with LC3-II required HBD of MAP1S because such interaction disappeared in cells without MAP1S (Fig. 7i) or expressing FL∆ (Fig. 7m).

**Fig. 8:**
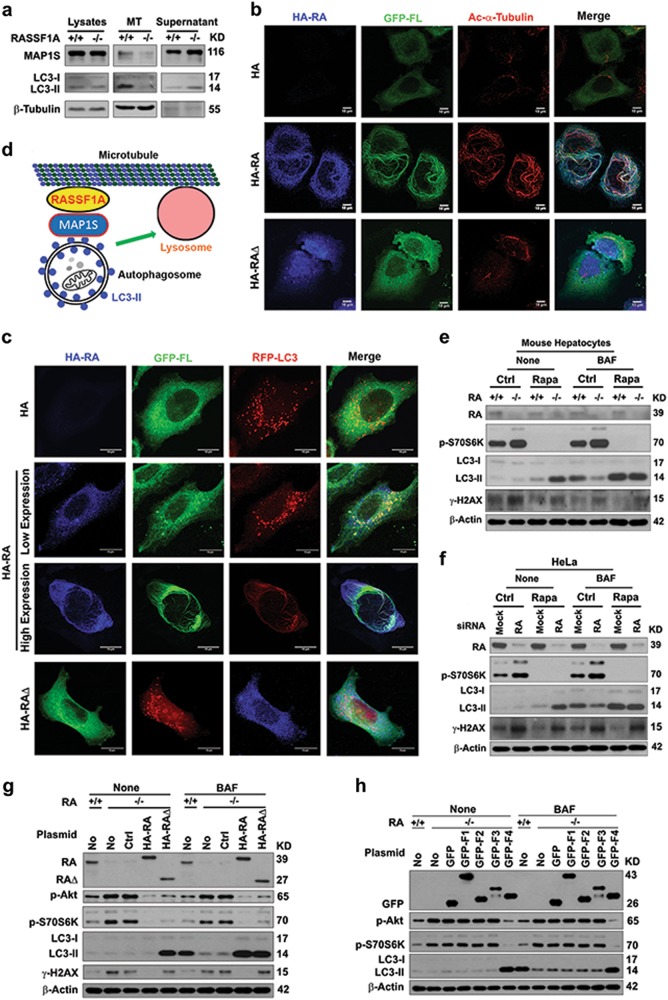
RASSF1A helps recruit autophagosomes onto acetylated microtubules through MAP1S and regulates autophagy initiation and autophagosomal degradation. **a** Representative immunoblots showing levels of MAP1S and LC3 associated with microtubules assembled in vitro. MT, assembled microtubules. β-Tubulin serves as the marker for assembled microtubules. **b**, **c** Representative fluorescent images showing the impact of RASSF1A (blue) on the distribution of GFP-FL MAP1S (green) and acetylated α-tubulin (red) (**b**) or RFP-LC3 (red) (**c**) in HeLa cells transiently expressing HA-tagged RASSF1A and GFP-tagged FL (**b**) or HA-tagged RASSF1A, GFP-tagged FL and RFP-LC3 (**c**). **d** A diagram showing the mechanism by which RASSF1A promotes autophagosomal trafficking. **e**, **f** Representative immunoblots showing the levels of p-S70S6K, LC3, and γ-H2AX in hepatocytes isolated from wild-type and RASSF1A^−/−^ mice (**e**) or HeLa cells treated with random (Mock) and RASSF1A-specific siRNAs (RA) (**f**) untreated (Ctrl) or treated with rapamycin (Rapa) in the absence (None) or presence of bafilomycin A1 (BAF). **g** Representative immunoblots showing the levels of p-Akt, p-S70S6K, LC3, and γ-H2AX in wild-type HeLa cells (+/+) or HeLa cells with RASSF1A knockout (−/−) transiently transfected with no plasmid (No), control vector (Ctrl), HA-RA or HA-RA∆ in the absence (None) or presence of bafilomycin A1 (BAF). **h** Representative immunoblots showing the levels of p-Akt, p-S70S6K and LC3 in wild-type HeLa cells (+/+) or HeLa cells with RASSF1A knockout (−/−) transiently transfected with no plasmid (No), control vector (GFP), GFP fused fragments in the absence (None) or presence of bafilomycin A1 (BAF)

To understand whether the interactions among RASSF1A, MAP1S and LC3-II enabled them to associate with microtubules, we first conducted in vitro microtubular assembling assays. RASSF1A enabled MAP1S and LC3-II to associate with microtubules while much less MAP1S and LC3-II was associated with microtubules assembled from lysates from RASSF1A^−/−^ hepatocytes (Fig. 8a). RASSF1A helped recruit MAP1S and then LC3 on RASSF1A-stabilized acetylated microtubule, while a RASSF1A mutant with the MAP1S and LC3-interactive RA domain deleted (RA∆) was unable to recruit MAP1S and LC3 to microtubular fibrils (Fig. 8b, c). Low levels of RASSF1A enabled MAP1S-associated LC3-labeled autophagosomal punctate foci to bind with less-stable microtubules while high levels of RASSF1A forced the autophagosomes to align with the frozen microtubule fibrillar structure (Fig. 8c). Taken together, RASSF1A enhances microtubular acetylation and recruits autophagosomes onto microtubules for trafficking through the LC3-interactive MAP1S (Fig. 8d).

To dissect the impact of RASSF1A on autophagy initiation and maturation, we treated hepatocytes isolated from wild-type and RASSF1A^−/−^ mice and Hela cells treated with random and RASSF1A-specific siRNAs with rapamycin, an inhibitor of mTOR signal. Rapamycin treatment completely inhibited mTOR activity as reflected by the levels of p-S70S6K either in the absence or presence of RASSF1A, enhanced autophagy initiation and maturation and autophagy flux in the presence of RASSF1A, and enhanced autophagy initiation but block autophagosomes degradation in the absence of RASSF1A; Consequently, rapamycin treatment enhanced autophagy flux and reduced levels of γ-H2AX in the presence of RASSF1A but impaired autophagy flux and promoted levels of γ-H2AX in the absence of RASSF1A (Fig. 8e, f). We observed no impact on autophagy flux when RASSF1A was overexpressed in HeLa cells (data not shown). We reasoned that levels of endogenous RASSF1A in those cells were already too high to be affected by the overexpressed RASSF1A. We generated RASS1A-knockout HeLa cells in which overexpressing RASSF1A did lead to activation of autophagy flux (Fig. 8g). The RASSF1A depletion-caused activation of mTOR signals were reduced in cells expressing either RA or RA∆; the RASSF1A depletion-caused inhibition of autophagy flux and increase of γ-H2AX levels were recovered by re-expressing RASSF1A; and the re-expression of RA∆ only caused the re-activation of autophagy initiation but not the re-activation of degradation of autophagosomes, leading to an accumulation of autophagosomes and maintenance of high γ-H2AX levels in the absence of bafilomycin A1 (Fig. 8g). The activation of mTOR and inhibition of autophagy initiation caused by RASSF1A depletion were recovered by expressing fragment F4, the SARAH domain of RASSF1A (Fig. 8h). Therefore, RASSF1A regulates both autophagy initiation and maturation through different domains to suppress genome instability and tumorigenesis.

## Discussion

Mammalian cells primarily use the autophagy-lysosome system to degrade dysfunctional organelles, misfolded/aggregated proteins and other macromolecules and maintain cellular homeostasis [4]. Autophagy defects lead to an enhancement of oxidative stress [4, 11]. Reactive oxygen species cause telomere attrition and DNA double strand breakage [39, 40] and simultaneously subvert mitotic checkpoints [41, 42]. The resulting genome instability is amplified through a cascade of autocatalytic karyotypic evolution through continuous cycles of chromosomal breakage-fusion-bridge and eventually leads to tumorigenesis [11, 43]. Furthermore, oxidative stress in turn activates NLRP3 inflammasomes that result in direct activation of caspase-1 [44]. Activation of caspase-1 eventually induces an inflammatory form of cell death referred to as pyroptosis [45]. The release of immunogenic danger signals or danger-associated molecular patterns (DAMPs) from pyroptotic cells can fuel pro-inflammatory cascades that promote the mortality of host structural, hematopoietic and immune-competent cells [46, 47]. Therefore, activating autophagy flux leads to suppression of cancer initiation and development, as well as the survival of cancer patients.

Autophagy initiation is regulated through the canonical PI3K-AKT-mTOR pathway or the non-canonical LKB1-AMPK-mTOR pathway [5]. Because RASSF1A interacts with MAP1S that regulates autophagy initiation through the non-canonical pathway [19, 23], we expected that RASSF1A would regulate autophagy initiation through the same pathway. However, RASSF1A did not alter the acetylation and stability of MAP1S. Overexpressing P27 did not restore the reduced autophagy flux caused by RASSF1A deletion, suggesting that RASSF1A did not regulate autophagy initiation in the same way as MAP1S does. RASSF1A interacts with MST1, one of the key regulators of the Hippo pathway that regulates organ size and apoptosis [35]. Conventionally, phosphorylated MST1 activates the phosphorylation of its downstream effector YAP so that YAP is retained in the cytoplasm and cannot enter the nuclear compartment to turn on the transcription of *CTGF* gene and other YAP target genes [35]. It was reported that RASSF1A stabilizes MST1 by preventing the dephosphorylation of MST1 by PP2A [20]. As expected, RASSF1A deletion causes a significant reduction in levels of total and phosphorylated MST1. However, RASSF1A deletion has no impact on levels of total and phosphorylated YAP and CTGF. Therefore, RASSF1A regulation of autophagy flux is not through the conventional Hippo pathway. It was reported that MST1 interacts with Beclin 1 and Bcl-2 and prevents autophagy initiation [35]. RASSF1A may regulate autophagy initiation through the MST1-Beclin 1-Bcl-2 pathway. We show here that RASSF1A depletion causes a reduction in levels of total and phosphorylated MST1, which predicts autophagy would be activated. Our results indicate that autophagy was not activated because of the reduction in levels of MST1 but actually inhibited. Therefore, RASSF1A did not regulate autophagy initiation in liver tissues through the MST1-Beclin 1-Bcl-2 pathway. It is known that MST1 interacts with AKT, leading to the activation of AKT kinase activity [36]. Activation of AKT induced by RASSF1A deletion leads to the activation of downstream effector of mTOR phosphorylated S70S6K to block autophagy initiation. We provide here an alternative mechanism by which MST1 regulates autophagy initiation through PI3K-AKT-mTOR pathway by direct binding with AKT. RASSF1A associates with mitochondria when microtubules are depolymerized [19] and colocalizes with MAP1S on stabilized microtubule, and RASSF1A and MAP1S distribute in different locations before they meet with each other on microtubules to promote autophagy maturation. Therefore, RASSF1A and MAP1S may regulate autophagy initiation through different mechanisms.

At the initial stages of autophagy, RASSF1A and MAP1S may regulate autophagy by their respective mechanisms. After autophagy is initiated, formed autophagosomes need to travel to fuse with lysosomes in different subcellular location to form autolysosomes [15, 37]. Then RASSF1A recruits LC3-II-associated autophagosomes through MAP1S on its stabilized acetylated microtubules where autophagosomes are delivered to fuse with lysosomes to form autolysosomes in which the captured substrates including LC3-II are degraded [6]. Microtubules are filamentous intracellular structures made of α-tubulin and β-tubulin heterodimers and other microtubule-associated proteins. Microtubules are constantly engage in cycle of polymerization and de-polymerization which play critical roles in the attachment and de-attachment of their transported cargos but also in cellular migration [6]. Without RASSF1A, formed autophagosomes are not recruited to microtubules to become matured to autolysosomes, which will lead to an accumulation of protein aggregates and dysfunctional organelles such as mitochondria and lipid droplets to induce oxidative stress and cell death including apoptosis and pyroptosis. When there is too much RASSF1A, microtubules remain in their acetylated state and become frozen so that progression of the cell cycle is blocked, cells are not able to migrate and cells die as others reported [15, 16, 18, 19]. Therefore, exploring how RASSF1A is regulated in cells will help to understand the polymerization/de-polymerization cycle of microtubules and needs to be further investigated.

RASSF1A activates autophagy initiation and facilitates autophagosomal trafficking so that autophagy flux is dramatically reduced when RASSF1A is depleted. Reduction in autophagy flux leads to oxidative stress and genome instability both of which promote the initiation and development of HCC. Autophagy is closely related to mouse lifespans [24, 29]. RASSF1A knockout mice carrying DEN-induced HCC live much shortened lifespans than the wild-type suffering with HCC possibly also due to autophagy defects. We recently reported that enhancing autophagy flux with spermidine prevents HCC and prolongs mouse lifespan [24]. Consistent with that observation, RASSF1A enhances autophagy flux to suppress HCC and improve survivals of mice suffering from HCC.
